# Significant Association of Interleukin4 Intron 3 VNTR Polymorphism with Susceptibility to Gastric Cancer in a South Indian Population from Telangana

**DOI:** 10.1371/journal.pone.0138442

**Published:** 2015-09-18

**Authors:** Amar Chand Bhayal, Devulapalli Krishnaveni, Kondadasula Pandu Ranga Rao, Adi Rakesh Kumar, Akka Jyothy, Pratibha Nallari, Ananthapur Venkateshwari

**Affiliations:** 1 Institute of Genetics and Hospital for Genetic Diseases, Osmania University, Begumpet, Hyderabad, India; 2 Department of Gastroenterology, Osmania General Hospital, Hyderabad, India; 3 Department of Genetics, Osmania University, Hyderabad, India; The Ohio State University, UNITED STATES

## Abstract

**Background:**

Gastric cancer (GC) is the fifth most common malignancy and remains a considerable public health burden worldwide. Genetic variations in genes encoding cytokines and their receptors influence the intensity of the *Helicobacter pylori* associated inflammatory response, which may contribute to individual differences in the outcome and severity of the disease. Interleukin4 is a typical pleiotropic T helper 2 (Th2) cytokine and is a critical mediator of Th1/Th2 balance. It is involved in the regulation of inflammation-mediated carcinogenesis in human organs, including gastric cancer.

**Objective:**

The present retrospective case control study was undertaken to evaluate the association of IL4 intron 3 VNTR polymorphism with the susceptibility to GC in a south Indian population from Telangana state.

**Materials and Methods:**

A total of 182 patients with diagnosed GC and 326 randomly selected healthy controls were enrolled in the present study. Genomic DNA was extracted from peripheral leukocytes and genotyping was determined by PCR-based assay. Association between genotypes and gastric cancer was examined by unconditional logistic regression analysis.

**Result:**

The variant 3R/2R and 2R/2R genotypes of IL4 exon3 VNTR polymorphism had about 1.9 fold and 3fold increased GC risk, respectively, when compared with 3R/3R genotype [3R/2R *vs*. 3R/3R: adjusted odds ratio (AOR) = 1.90, 95% confidence interval (CI) = 1.23–2.95 P = 0.004 and 2R/2R *vs*. 3R/3R: AOR (95%CI) = 2.96 (1.29–6.82), P = 0.011]. Furthermore, a significant increased risk of GC was found for the 2R allele carriers (3R/2R + 2R/2R) compared with the 3R/3R genotype (AOR (95%CI) = 2.04 (1.35–3.10), P = <0.000). The IL4 2R allele frequency was 0.28 among the GC group and 0.18 among the controls, and the difference was statistically significant (P = <0.000).

**Conclusion:**

The present study revealed an association of 2R allele and 2R carrier genotypes in the etiopathogenesis of GC in south Indian population.

## Introduction

Despite declining incidences and mortality rates observed worldwide, gastric cancer (GC) is the fifth most common cancer (952,000 cases, 6.8% of the total) and ranks third as the cause of cancer related mortality (723,000 deaths, 8.8% of the total), [1]. Its pattern and incidence rates show considerable variation according to age, gender, ethnicity, socio-economic conditions and geographical location across the world [2]. More than 70% of cases occur in developing countries and half the world total occurs in Eastern Asia. Age-standardized incidence rates are about twice as high in men as in women in most of the studied populations [3]. Annual incidence rate of gastric cancer in India is 10.6 per 100 000 population. The incidence rate of gastric cancer is four times higher in southern India compared with northern India. Gastric cancer is the third most common cancer in southern India [4].

Like most other cancers, GC has a multifactorial and multistep etiology that involves complex interplay among *Helicobacter pylori* (HP) infection, exogenous environmental and endogenous genetic factors [5]. Persistent *Helicobacter pylori* infection, leading to chronic inflammation, plays a major role in gastric carcinogenesis and is preceded by a lengthy precancerous process, developing via multiple sequential steps [6].

Interleukins (ILs) help mediate many of the effector phases of immune and inflammatory response [7]. IL4 is a prominent anti-inflammatory prototypic Th2 type cytokine and plays a key role in activation and differentiation of B cells and mast cells, antibody production and development of the Th2 subsets of lymphocytes [8]. IL4 is secreted by a variety of cells, such as: T cells, mast cells, antigen presenting cells and NK cells, etc. It is a potent down regulator of macrophage function, inhibits the secretion of proinflammatory cytokines such as interferon-γ, IL1, IL6, and tumor necrosis factor α (TNFα) [9].

The IL4 gene is located on the long arm of chromosome 5 (q31.1) together with other Th2 cytokine genes and is present in a cluster of cytokine genes (IL-3, -5, -9, -13, and -15, granulocyte colony-stimulating factor, and interferon regulatory factor) [10]. IL4 gene has 4 exons and is approximately 10 kb in size. Common polymorphisms in IL4 reported by various studies are: –590C/T (rs2243250) in promoter region, –33C/T (rs2070874), –168G/C (rs2070874) in the 5ꞌ untranslated region and VNTR polymorphism in intron3. A variable number of tandem repeat (VNTR) of 70 base pair repeat is situated in third intronic region of the IL4 gene. Three repeat (3R) allele is more common and two repeat (2R) allele is relatively rare. There is another rarer allele of four repeat, which is reported in only a few populations [11]. Two repeat (2R) allele was found to be a high producer of IL4 [12].

Keeping in view the importance of IL4 in local and systemic anti inflammatory effects, the present study is aimed to evaluate the association of IL4 VNTR polymorphism with GC in our population. We also examined whether the potential association of this polymorphism with gastric cancer risk differs with regard to demographic features.

## Materials and Methods

### Study Population

A total of 508 subjects were enrolled in the present study, 182 patients with GC and 326 healthy control subjects. Gastric cancer patients were recruited from the Department of Gastroenterology, Osmania General Hospital, Hyderabad. Gastric cancer patients, who were diagnostically confirmed through upper gastrointestinal endoscopy (UGIE) and histopathological examination during the study period between Nov. 2009 and Oct. 2013, were considered for the present study. Healthy ethnicity matched controls were selected randomly from a similar geographical region to that of the patients. The selection criteria for the controls included no individual history of cancer and the exclusion criteria were past or present gastric ulcer, immunosuppressive disorders and other major systemic diseases. A structured questionnaire was used to elicit information on epidemiological factors such as age, sex, dietary habits, addictions, family history of cancer etc. The study protocol was approved by Research Ethics committee of Institute of Genetics and Hospital for Genetic Diseases (Osmania University, Hyderabad) and informed written consent was obtained from all recruited subjects. The scientific investigation presented in this paper has been carried out in accordance with the Code of Ethics of the World Medical Association (Declaration of Helsinki) for experiments involving humans.

### Sample collection

Approximately 5 ml of peripheral blood from each subject was collected into EDTA coated vacutainers for subsequent DNA extraction and *H*. *pylori* serology. Once processed, whole blood and plasma samples were aliquoted and stored at –20°C until analysis. The genomic DNA was extracted from peripheral blood leukocytes using the salting-out method as previously described [13].

### Detection of *Helicobacter pylori* Infection

*H*. *pylori* status was assessed by serologic analysis. The antiH. pylori IgG antibody titer was determined by ELISA according to the manufacturer’s protocol (IBL International, GMBH, Germany).

### Genotyping of IL4 VNTR polymorphism

IL-4 variable number of tandem repeat (VNTR) was amplified through PCR based assay, using forward primer, 5’-TAGGCTGAAAGGGGGAAAGC-3ꞌ and reverse primer, 5’-CTGTTCACCTCAACTGCTCC-3’ [14]. PCR was carried out in a volume of 10 μl containing 2μl (20–40 ng) of genomic DNA, 1X reaction buffer, 0.125 mM deoxynucleotide triphosphates(dNTPs), 1.5 mM MgCl_2_, 0.60 μM of each primer and 0.3 units of Taq DNA polymerase (Bangalore Genei). The PCR protocol was: initial denaturation at 95°C for 7 minutes, followed by 35 cycles at 95°C for 45 seconds, 56°C for 45 seconds, and 72°C for 45 seconds and a final extension at 72°C for 7 minutes. The PCR products were resolved by electrophoresis on 3% agarose gel stained with ethidium bromide. The size of the amplified products was directly diagnostic of number of repeats in the intervening sequence. Alleles were named as follows: allele 3R = three repeats (253 bp), and allele 2R = two repeats (183 bp). The alleles were named as 3R and 2R to minimize the confusion as other papers have named the same alleles as 1 and 2 or B1 and B2 or RP1 and RP2. For quality control, 10% of randomly selected samples containing both cases and controls were analyzed a second time without finding any discrepancies.

### Statistical analysis

To analyze the demographic characteristics, we used the Mann–Whitney U test and univariate logistic regression for continuous and categorical data, respectively. Age was categorized into three groups (i.e.≤45 yrs, 46–60 yrs and > 60yrs). For smokers, the number of pack-years smoked was calculated to indicate the cumulative smoking dose [pack-years = (cigarettes per day/20) * (years smoked)]. Moderate and chronic smokers were categorized by using the median pack-year value (25 Pack years) as the cut points. Information collected on the frequency of alcohol use per week and the total duration (in years) of alcohol consumption was used to derive drinking frequency-years [(times of drinking-per week)*(years drinking)]. Alcoholics were divided into moderate and chronic groups by using the median frequency-years as the cut points. Deviations from the Hardy–Weinberg equilibrium (HWE) were tested using the x^2^ goodness-of-fit test. Unconditional multiple logistic regression model was used to calculate the adjusted odds ratios (ORs) and corresponding 95% confidence intervals (CIs) after controlling for age, gender, smoking, tobacco chewing and alcohol consumption. Analysis for the genotypes was done under co-dominant, recessive, dominant and log-additive models of inheritance. The Akaike information criterion (AIC) was used to determine the best-fit model. All the statistical tests were two sided, and were considered significant at P value ≤0.05. Statistical analysis was performed by R version 3.1.2 [15] and R package “SNPassoc” [16].

## Results

### Characteristics of the patients and controls

Epidemiological characteristics of patients and controls are presented in Table 1. The mean age of the GC patients and that of the controls was 52.66±12.15 (range = 23–83; median = 55) and 50.15±12.77 (range = 23–80; median = 52) years, respectively with significant difference between two groups (p- value = 0.026). There was about 1.65 and 1.67 fold increased risk of GC for middle and higher age groups respectively, compared to lower age group. Male preponderance was found with about 1.8 fold increased risk of GC in males. The smokers in the controls and GC groups were 36.5% and 59.3% respectively and there was 2.5 fold increased risk for smokers when compared to nonsmokers (OR = 2.54 *P*<0.000). When divided into moderate and chronic smokers there was 2- and 3.7- fold increased risk of GC, respectively with reference to nonsmokers. Tobacco chewers were at about 4 fold increased risk of GC when compared with nonchewers. Similarly with respect to alcohol consumption, we found significant difference with overall alcoholic group (~2.2fold) as well as with moderate (1.9 fold) and chronic (2.9 fold) alcoholic groups when compared to nonalcoholic group.

**Table 1:** Distribution of Epidemiological characteristics in GC Patients and Controls.

Epidemiological characteristics	Control (%) (N = 326)	GC (%) (N = 182)	Crude OR (95%CI)	P-Value
**Age (Mean±SD)**	50.15±12.77	52.66±12.15		**0.026^a^**
**Age Group**				
** ≤45 Years**	134(41.1)	54(29.7)	1.00 (ref)	
** 45–60 Years**	119(36.5)	79(43.4)	1.65(1.08–2.52)	**0.021**
** >60 Years**	73(22.4)	49(26.9)	1.67(1.03–2.69)	**0.037**
** Sex**				
**Female**	139(42.6)	54(29.7)	1.00 (ref)	
**Male**	187(57.4)	128(70.3)	1.76(1.20–2.58)	**0.004**
**Smoking**				
**Non smokers**	207(63.5)	74(40.7)	1.00 (ref)	
**Smokers**	119(36.5)	108(59.3)	2.54(1.75–3.68)	**<0.000**
** Moderate smokers**	82(25.2)	59(32.4)	2.01(1.31–3.09)	**0.001**
** Chronic smokers**	37(11.3)	49(26.9)	3.71(2.24–6.12)	**<0.000**
**Tobacco chewing**				
**No**	238(73.0)	73(40.1)	1.00 (ref)	
**Yes**	88(27.0)	109(59.9)	4.04(2.75–5.93)	**<0.000**
**Alcoholism**				
**Non alcoholic**	204(62.6)	79(43.4)	1.00 (ref)	
**Alcoholic**	122(37.4)	103(56.6)	2.18(1.51–3.15)	**<0.000**
** Moderate alcoholic**	89(27.3)	66(36.3)	1.92(1.27–2.89)	**0.002**
** Chronic alcoholic**	33(10.1)	37(20.3)	2.90(1.69–4.95)	**<0.000**

OR:Odds ratio, CI:confidence interval, ref: reference

a: Mann-Whitney Test

### Analysis of IL4 VNTR polymorphism

The genotypic and allelic frequencies of the IL-4 intron 3 VNTR polymorphism in patients with GC and controls are shown in Table 2. Genotype analysis revealed no significant deviation from the HWE in the patients and control groups (p values: 0.320 in cases, 0.141 in controls, and 0.489 in both). The frequency of 3R/3R, 3R/2R, and 2R/2R genotype was 53.30 versus 69.02%, 36.36 versus 26.69%, and 9.18 versus 4.29%, in the GC patient group as compared to the control group respectively. To determine the association between genetic polymorphism and GC, multiple logistic regression analysis was applied. Multivariate logistic regression analysis revealed that subjects with 3R/2R and 2R/2R polymorphic genotypes had a significantly higher risk of 1.9 fold (95% CI = 1.23–2.95; p- value = **0.004**) and 2.96 fold (95% CI = 1.29–6.81; p- value = **0.011**) risk of having GC, when compared with the 3R/3R *i*.*e*. *major* genotype. Likewise, there was significant increase in frequency of 2R alleles [OR (95%CI) = 1.82(1.34–2.47); p-value = **0.00015**] in GC patients group in comparison to the control group. When we combined 3R/2R and 2R/2R genotypes, assuming a dominant allele effect, the combined (3R/2R +2R/2R) variant genotypes were associated with 2.04 fold (95% CI = 1.35–3.10; p-value = **<0.000**) increased risk of GC for 2R allele carriers. Based on the values of Akaike information criterion (AIC), dominant model with least AIC value was found to be best genotypic model indicating about 2 fold increased risk for 2R allele carriers.

**Table 2:** Distribution of Genotypes and allele frequencies of VEGF -2549 I/D polymorphism in Gastric cancer patients and Control subjects.

Genotypes/Alleles	Control (N = 326)	Gastric Cancer(N = 182)	Crude OR (95%CI);P-Value	Adjusted OR (95%CI)	Adjusted P-Value	AIC
**Co-Dominant**						
3R/3R	225(69.02)	97(53.30)	1.00(Ref.)	1.00(Ref)		
3R/2R	87 (26.69)	68(36.36)	1.81(1.22–2.69); 0.003	1.90(1.23–2.95)	**0.004**	
2R/2R	14 (4.29)	17(9.18)	2.82(1.34–5.94); 0.007	2.96 (1.29–6.81)	**0.011**	590.3
**Alleles**						
3R	537(0.82)	262(0.72)				
2R	115(0.18)	102(0.28)	1.82(1.34–2.47); 0.00015	1.80 (1.29–2.51)^¶^	**<0.000^¶^**	588.5^¶^
**Dominant Model**						
3R/3R+3R/2R(vs.3R/3R)	101(30.98)	85 (46.70)	1.95 (1.34–2.84); <0.0001	2.04 (1.35–3.10)	**0.0007**	589.4
**Recessive Model**						
2R/2R (vs.3R/ 3R+3R/2R)	14 (4.29)	17 (9.34)	2.29(1.10–4.78); 0.026	2.33 (1.03–5.26)	**0.0407**	596.6

AIC: Akaiki information criteria; OR: Odds ratio; CI: confidence interval; Ref = Reference

¶: Log-additive model values

### Association of genotypes with epidemiological features

We examined whether the associations of this polymorphism with gastric cancer risk were modified by interaction with other risk factors including age, gender, smoking, tobacco chewing and alcohol consumption. To maximize power in the cross-classification/interaction analysis, subjects with heterozygote (3R/2R) and homozygous (2R/2R) variant genotypes were combined and ORs were expressed with reference to the non variant genotype (3R/3R) and the first category of the covariates. Cross-classification analysis of IL4 VNTR polymorphism with demographic variables is presented in Table 3. Cross-classification analysis of IL4 VNTR polymorphism with age groups showcased marginally increased risk of GC for 2R carrier subjects who were middle aged and older (4.08 fold vs. 3.38fold) when compared with lower age group / major genotype carrier subjects. Cross-classification analysis with sex showed marginally increased risk of GC in 2R allele carrier female subjects than male subjects (2.87 fold vs. 2.52 fold). Similarly there was more increased risk to 2R allele carrier chronic smokers than moderate smokers and non smokers when compared with major genotype carrier nonsmokers. Chronic alcoholic 2R carrier subjects were at more pronounced risk than moderate alcoholics (3.54 fold vs. 2.53 fold) compared to major genotype carrier nonalcoholic subjects. However, all p-interaction values were statistically insignificant indicating lack of interaction.

**Table 3:** Cross-classification analysis of IL4 VNTR polymorphism with Epidemiological covariates.

**Cross-classification analysis with covariate Age group**									p-interaction: 0.35		
	**≤45 Years**			**46–60 Years**				**>60 Years**			
**Genotypes**	**Control**	**GC**	**OR (95% CI)**	**Control**	**GC**		**OR (95% CI)**	**Control**		**GC**	**OR (95% CI)**
**3R/3R**	79	27	1.00 (Ref)	92	39		1.37(0.73–2.54)	54		31	2.03 (1.04–3.96)
**3R/2R-2R/2R**	55	27	1.56 (0.80–3.08)	27	40		4.08 (2.01–8.29)	19		18	3.38 (1.46–7.86)
**Cross-classification analysis with covariate Sex**								p-interaction: 0.25			
	**Female**						**Male**				
**Genotypes**	**Control**		**GC**	**OR (95% CI)**			**Control**	**GC**	**OR (95% CI)**		
**3R/3R**	103		28	1.00 (Ref)			122	69	1.82 (0.75–4.42)		
**3R/2R-2R/2R**	36		26	2.87 (1.41–5.87)			65	59	2.52 (1.33–4.80)		
**Cross-classification analysis with covariate Smoking**								p-interaction: 0.99			
	**Nonsmoker**			**Moderate Smoker**				**Chronic Smoker**			
**Genotypes**	**Control**	**GC**	**OR (95%CI)**	**Control**		**GC**	**OR (95% CI)**	**Control**	**GC**		**OR (95% CI)**
**3R/3R**	153	42	1.00(Ref)	47		27	1.09 (0.50–2.38)	25	28		2.08 (0.96–4.51)
**3R/2R-2R/2R**	54	32	2.02 (1.11–3.68)	35		32	2.17 (1.00–4.72)	12	21		4.51 (1.82–11.22)
**Cross-classification analysis with covariate Tobacco chewing**									p—interaction: 0.61		
	**No**						**Yes**				
**Genotypes**	**Control**	**GC**		**OR (95% CI)**			**Control**	**GC**	**OR (95% CI)**		
**3R/3R**	163	39		1.00(Ref)			62	58	2.92 (1.26–6.75)		
**3R/2R-2R/2R**	75	34		1.85 (1.06–3.24)			26	51	6.04 (3.13–11.67)		
**Cross-classification analysis with covariate Alcoholism**								p-interaction: 0.85			
	**Nonalcoholic**			**Moderate Alcoholic**				**Chronic Alcoholic**			
**Genotypes**	**Control**	**GC**	**OR (95% CI)**	**Control**		**GC**	**OR (95% CI)**	**Control**	**GC**		**OR (95% CI)**
**3R/3R**	150	49	1.00(Ref)	53		28	1.09 (0.52–2.26)	22	20		1.53 (0.68–3.44)
**3R/2R-2R/2R**	54	30	1.82 (1.00–3.32)	36		38	2.53 (1.22–5.25)	11	17		3.54 (1.41–8.88)

OR: Odds ratio; CI: confidence interval; Ref = Reference. AOR adjusted for Sex, Age group, Tobacco chewing, Smoking and Alcoholism.

The association between polymorphism and gastric cancer stratified on demographic features like age, sex, tobacco chewing, smoking and alcohol use are presented in Table 4. Stratification analysis of demographic features with respect to genotypes revealed that risk of GC was more pronounced in subjects who carried 3R/2R or 2R/2R genotypes and were middle-aged(OR = 2.99(1.53–5.86)), females (OR = 2.87(1.41–5.87)), tobacco chewers (OR = 2.30 (1.24–4.28)) and alcoholics (OR = 2.89(1.50–5.55)).

**Table 4:** Stratification analysis of IL4 VNTR genotype frequencies, ORs and 95% CIs in Gastric cancer.

Demographical Features	Control(N = 326)			GC(N = 182)		AOR (95%CI)*
	3R/3R	3R/2R+2R/2R		3R/3R	3R/2R+2R/2R	
**Age Group**						
** ≤45 Years**	79	55		27	27	1.56(0.80–3.08)
** 45–60 Years**	92	27		39	40	**2.99 (1.53–5.86)**
** >60 Years**	54	19		31	18	1.67(0.71–3.88)
**Sex**						
** Female**	103	36	28		26	**2.87 (1.41–5.87)**
** Male**	122	65	69		59	**1.72 (1.04–2.85)**
**Smoking**						
** Non Smokers**	153	54		42	32	**2.02(1.11–3.68)**
** Smokers**	72	47		55	53	**2.04(1.14–3.64)**
** Moderate Smokers**	47	35		27	32	2.00(0.97–4.12)
** Chronic Smokers**	25	12		28	21	2.17(0.84–5.63)
**Tobacco Chewing**						
** No**	163	75		39	34	**1.85(1.06-3.24)**
** Yes**	62	26		58	51	**2.30(1.24–4.28)**
**Alcoholism**						
** Non Alcoholic**	150	54		49	30	**1.85(1.01–3.36)**
** Alcoholic**	75	47		48	55	**2.89(1.50–5.55)**
** Moderate Alcoholic**	53	36		28	38	**2.31(1.15–4.62)**
** Chronic Alcoholic**	22	11		20	17	2.25(0.79–6.38)

AOR: Adjusted Odds ratio; CI: confidence interval

*AORs were adjusted for age, sex, smoking, Tobacco chewing, and alcohol consumption in a logistic regression model.

## Discussion

In the present study, we investigated the association between IL4 VNTR gene polymorphism and risk of gastric cancer in a South Indian population. The most important result of our study was the significant association of 2R allele and 2R carrier genotypes (i.e. 3R/2R or 2R/2R) with increased risk of GC, indicating the possible association of IL4 VNTR polymorphism with GC. To the best of our knowledge, this is the first association study that attempted to evaluate the association of IL-4 gene VNTR polymorphism on GC in a south Indian population.

GC is a classical example of inflammation induced malignancy. IL4 is an important anti inflammatory cytokine which inhibits *H*. *pylori*-induced gastric mucosal inflammation and atrophy by decreasing interferon γ (IFN-γ) and other Th1-type cytokines [17]. IL4 induces immature effector cells to assume a Th2 phenotype and also represses Th1-inducing signals. A balance between Th1 and Th2 cytokines by IL-4 therefore crucially influences the outcome of *H*. *pylori* infection. Th2 T-cell response, represented by IL4, plays a protective role in the development of gastric cancer. Further *IL-4*-deficient mice infected with *H*. *pylori* show severe gastric inflammation compared with wild-type mice [18]. In cancer cell lines, variations in the activity of IL4 and its receptor have been shown to modulate cell proliferation and to affect signal transduction pathways [19]. IL-4 is reportedly associated with cancer development *via* its suppression of inflammation and angiogenesis and directly inhibits the growth of human melanoma, renal cell carcinoma and gastric cancer cells [20]. IL4 has further been described to cause a dose dependent reduction of proliferation [21] and to inhibit matrix metalloproteinases (MMP-1, -2 and -9), cell matrix invasion and cell migration [22]. IL-4 may also inhibit the cell-mediated immune response by downregulating the expression of Th1 cytokines, such as IFN- γ and IL-2, and by decreasing the quantity and quality of the CD8+ T-cell response in the tumour microenvironment.2R allele has been shown to enhance the IL-4 production or activity by T cells [12]. Thus it is possible that higher IL4 production might result in the escape of tumour cells from immune surveillance due to diminished cell mediated immune response.

Several epidemiological studies have examined the association between the IL4 VNTR polymorphism and the risk of cancer, including prostate, urothelial, breast, colorectal, and gastric cancers but the results are inconsistent. In a study by Yang et al (2014) IL-4 intron 3 VNTR polymorphism was found to be associated (2R/2R vs. 3R/3R+3R/2R, AOR = 1.46, 95% CI: 1.05–2.04) with early stage oral and pharyngeal carcinoma risk (OPSCC), which also interacted with alcohol consumption (p = 0.024) [23]. 2R carrier genotypes (i. e. 3R/2R and 2R/2R) were reported to be associated with late stage bladder cancer in a northern Indian population [24] (Ahirwar et al; 2008). In a case control study involving 138 patients with transitional cell carcinoma (TCC) of the urinary bladder and 105 healthy controls from Taiwanese population, 2R/2R (RP1/RP1) was found to be significantly associated (OR = 8.88 (1.02–77.16); *P* = 0.018) with bladder cancer and tumour invasiveness [25]. Shekari et al (2012) found no significant risk of developing cervical cancer with 2R/3R (RP1/RP2) genotypes in northern Indian women [26]. There was no association of IL4VNTR polymorphism with the risk of Prostate cancer in a northern Indian population but two fold risk with progression to bone metastasis in prostate cancer was reported [27]. No association of this polymorphism was found with the risk of breast cancer in a northern Indian population [28]. No significant differences in genotype distributions or allelic frequencies of the IL-4 gene intron3 polymorphism were observed between the oral cancer patients and controls in a study from Taiwanese population [29]. Similarly, in a case-control study involving 123 GC patients and 103 controls, Lai et al (2005) found no association of this polymorphism with the risk of GC in Taiwanese population [30]. In contrast the present study revealed an association between gastric cancer and IL4 VNTR polymorphism. To the best of our knowledge the present study is the first report regarding the positive association of interleukin-4 intron 3 VNTR polymorphism with the increased risk of GC.

A subject’s epidemiological/life style related factors may also play an important role in the etiology of GC and these factors may act in synergy with genetic polymorphism of IL4. As expected, addictions, such as tobacco chewing, smoking and alcohol use, were correlated with significantly increased susceptibility to GC. Further, the variant 2R carrier genotypes were found to exhibit more pronounced risk with respect to female sex, middle (45–60 years) age group, chronic smoking, tobacco chewing and chronic alcoholism indicating modulation of risk by these epidemiological factors. However, the numbers within each sub-group were too low and larger studies are warranted to exhibit significant interaction.

Our study has certain limitations which need to be considered in interpreting our results. Community based Control subjects were not matched for age and gender. Although MLR was applied, effect of residual confounding can not be completely ruled out. Our sample size was relatively small which may influence the statistical power in the study, particularly for interaction and stratified analysis. We did not have detailed Clinico-pathological information of all the patients which does not permit us to analyze our data in the context of histological subtypes, grades and stages of gastric cancer. Further in the light of the large repertoire of SNPs in pro- and anti-inflammatory cytokine genes with complex interactions in a multifactorial complex disease like gastric cancer, the results of this study may be insufficient to clarify the complex interplays among a number of different genes in GC but may represent a hypothesis generating data for further investigations.

## Conclusion

In conclusion our results suggest that variant genotypes and allele of IL 4 VNTR polymorphism may significantly increase the susceptibility to GC and implicates the 2R allele as one of the potential genetic markers in the etiology of the disease in our population. However, a large confirmatory study involving other populations is warranted to understand the population-specificity and the relative contribution of this polymorphism in the etiology of gastric cancer.
